# Severe thrombocytosis and anemia associated with celiac disease in a young female patient: a case report

**DOI:** 10.1186/1752-1947-2-96

**Published:** 2008-04-01

**Authors:** Wieland Voigt, Karin Jordan, Christoph Sippel, Mroawan Amoury, Hans-Joachim Schmoll, Hans H Wolf

**Affiliations:** 1 Department of Hematology/Oncology, Martin-Luther-University, Halle-Wittenberg, 06120 Halle/Saale, Germany; 2 Emergency Care Unit, Martin-Luther-University, Halle-Wittenberg, 06120 Halle/Saale, Germany

## Abstract

**Introduction:**

Platelet counts exceeding 1.000 × 10^3^/μl are usually considered secondary to another cause, particularly to chronic myeloproliferative disease (CMPD). Reactive thrombocytosis due to iron deficiency rarely exceeds platelet counts of 700 × 10^3^/μl.

**Case presentation:**

Here we report the case of a young woman presenting with clinical signs of severe anemia. Laboratory findings confirmed an iron-deficiency anemia associated with severe thrombocytosis of 1703 × 10^3^/μl. Macroscopic gastrointestinal and genitourinary tract bleeding was excluded. The excessive elevation of platelets, slightly elevated lactate dehydrogenase and slightly elevated leukocytes along with the absence of other inflammation parameters raised the suspicion of an underlying hematological disease. However, bone marrow evaluation could not prove the suspected diagnosis of a CMPD, especially essential thrombocythemia (ET). In the further clinical course the platelet count returned to normal after raising the hemoglobin to a level close to normal range with erythrocyte transfusion, and normalization of serum iron and decline of erythropoietin. Finally, following small bowel biopsy, despite the absence of typical clinical signs, celiac disease was diagnosed. After discharge from hospital the patient was commenced on a gluten-free diet and her hemoglobin almost completely normalized in the further follow-up period.

**Conclusion:**

This case illustrates the rare constellation of an extreme thrombocytosis most likely secondary to iron deficiency due to celiac disease. This represents, to the best of the authors' knowledge, the highest reported platelet count coincident with iron deficiency. A potential mechanism for the association of iron-deficiency anemia and thrombocytosis is discussed. Even in the presence of 'atypically' high platelets one should consider the possibility of reactive thrombocytosis. Extreme thrombocytosis could emerge in the case of iron deficiency secondary to celiac disease.

## Introduction

Numerous diseases or conditions can cause an elevated platelet count in peripheral blood. It may be secondary to, for example, infection or inflammation or, mainly in elderly patients, based on myeloproliferative diseases [1,2]. The broad range of differential diagnoses is summarized in Table 1. Thus, it is of particular importance to rule out the possibility of any kind of reactive or hereditary cause of thrombocytosis before making the diagnosis of essential thrombocythemia (ET). In 2001 the World Health Organization published revised criteria for the diagnosis of ET. Positive criteria are a sustained platelet count of at least 600 × 10^3^/μl and bone marrow biopsy showing a proliferation of mainly megakaryocytic lineage with increased numbers of enlarged, hyperlobated, mature megakaryocytes. In addition, one has to exclude an underlying polycythemia vera, chronic myelogenous leukemia, chronic idiopathic myelofibrosis, myelodysplastic syndrome and other reasons for reactive thrombocytosis, particularly iron deficiency or inflammation (Table 1) [2].

**Table 1:** Differential diagnosis for thrombocytosis

Typical causes of thrombocytosis
• Chronic myeloproliferative disorders such as CML, PV, ET
• Certain myelodysplastic syndromes such as 5q-syndrome
• Underlying or occult malignancy
• Chronic inflammatory or infectious disease
• Asplenia
• Drug induced (for example, Vincristine, ATRA, cytokines, growth factors)
• Secondary after hemolytic crisis or hemorrhage
• Iron deficiency

The causes of iron deficiency are diverse and span from chronic gastrointestinal or genitourinary tract bleeding, insufficient iron intake from the diet or impaired intestinal iron absorption. One possible underlying intestinal disease associated with impaired iron absorption is celiac disease [3,4]. Previously, celiac disease was usually considered in patients who had signs of malabsorption and signs of gastrointestinal dysfunction such as diarrhea or steatorrhea, weight loss or impaired development. Furthermore, it is often associated with multiple deficiencies of macro- and micro-nutrients [3]. It has only recently become clear that in addition to the classical form of celiac disease there is a broad span of atypical forms that sometimes even completely lack the typical signs of celiac disease [3,5]. Hematologic manifestations of celiac disease have been recently published by Halfdanarson et al [3]. These include thrombocytopenia, thrombocytosis, leukopenia, venous thromboembolism or anemia secondary to malabsorption of iron, folic acid and/or vitamin B12. The prevalence of anemia varies with different reports of values between 12% and 69% and might be the major presenting feature. The diagnosis of celiac disease is usually made through small bowel biopsies and testing for anti-endomysial or anti-tissue-transglutaminase antibodies [3].

## Case presentation

In November 2006 a 25-year-old Palestinian woman was admitted to our emergency care unit with symptoms of vertigo and fatigue. She was in overall reduced physical condition, her skin and mucous membranes were pale and she suffered from infantile cerebral paresis and mental retardation. Her vital parameters were stable and there were no signs of hemorrhagic shock. Physical examination was normal with the exception of a reduced body mass index of 15.26. There were no signs of macroscopic bleeding from the gastrointestinal or genitourinary tract. The patient's mother reported no weight loss over the past few years and normal caloric intake and no gastrointestinal problems. There were no signs of acute or chronic inflammation.

**Figure 1:**
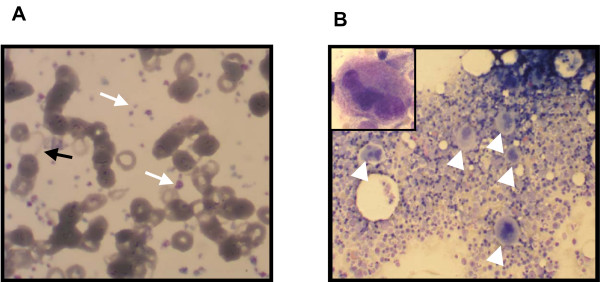
**Representative photographs of peripheral blood smear and bone marrow smear**. (A) In the peripheral blood smear there is an obviously increased platelet count with the presence of micro- and macro-platelets (white arrows). Erythrocytes reveal an anisocytosis and pronounced anulocytosis (black arrow). Note the presence of regular erythrocytes which represents the erythrocyte population after successful transfusion of two erythrocyte concentrates. (B) In the bone marrow smear there is a clearly increased count of juvenile megakaryocytes (white arrows). A representative megakaryocyte is shown at higher magnification in the inlet. No clustering of megakaryocytes is evident in this section.

Laboratory tests at admission revealed a marked microcytic hypochromic anemia with a hemoglobin level of 4.6 g/dl (normal range 11.68–15.84 g/dl), MCV 58 fl (normal range 85–95 fl) and a severe thrombocytosis of 1703 × 10^3^/μl (normal range 140–440 × 10^3^/μl). Leukocyte count was slightly elevated at 11.45 × 10^3^/μl (normal range 3.8–9.8 × 10^3^/μl) and differential blood count was regular. However, in her blood smear there was anisocytosis and reduced fraction of reticulocytes. Platelet morphology was conspicuous with microcytic and macrocytic forms (Figure 1A). Ferritin was below the measurable range (< 1.5 ng/ml), serum iron was 0.4 μmol/l (normal range 8.0–26 μmol/l), transferrin was 3.8 g/l (normal range 2.0–3.6 g/l) and transferrin saturation was 0.4% (normal range 16.0–45%). Lactate dehydrogenase was slightly elevated at 4.75 μmol/l (normal values below 4.12 μmol/l), folic acid was in the normal range and vitamin B12 was slightly reduced at 112 pmol/l (normal range 133–675 pmol/l). Strikingly, erythropoietin was strongly elevated at 854 mU/ml (normal range 4.28–29.50 mU/ml). Other routine laboratory parameters, in particular C-reactive protein and blood sedimentation, were within normal ranges.

As the patient presented with severe microcytic iron-deficiency anemia, we performed diagnostic investigations to rule out chronic bleeding with endoscopy of the upper and lower gastrointestinal tract and gynecological examination, all with negative results. Biopsies were taken. The patient underwent blood transfusion with regular increases in hemoglobin, which remained stable in the further course. Since the patient presented initially with a slightly elevated lactate dehydrogenase, extreme thrombocytosis and severe anemia with reduced reticulocytes in addition to excessive elevated erythropoietin, we performed further diagnostic investigations including ultrasound examination of the abdomen and a bone marrow biopsy to rule out a myeloproliferative syndrome, in particular ET. The ultrasound finding was normal with the exception of a slightly enlarged spleen that was 12 cm in longitudinal diameter. On the bone marrow smear, megakaryopoesis was significantly elevated. There were juvenile megakaryocytes which sometimes appeared to be clustered. Few megakaryocytes were hyperlobated. Erythopoesis was judged to be increased and not dysplastic. An iron stain revealed clearly decreased storage iron. Other findings were normal. Notably, the typical signs of myeloproliferative disease syndrome such as basophilic or eosinophilic precursors were absent. Overall, the bone marrow smear as well as the histological evaluation of the bone marrow biopsy did not support the possible diagnosis of ET (Figure 1B). Cytogenetic analysis of the bone marrow did not show any abnormalities.

**Figure 2:**
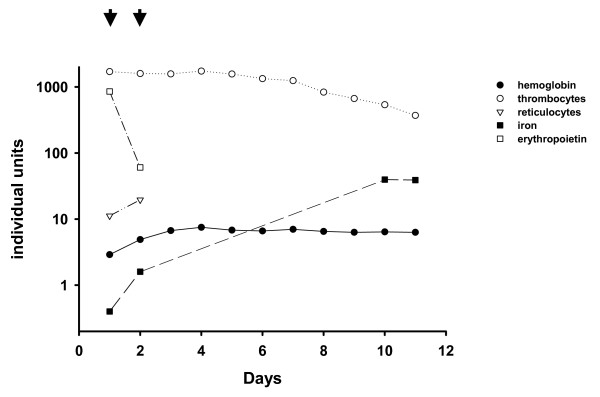
**Course of hematological parameters**. This figure summarizes the results of relevant laboratory testing. Clearly, concomitant with the transfusion of a total of erythrocyte concentrates (each arrow indicates two concentrates) there was a significant increase in hemoglobin, reticulocytes and serum iron. In parallel, the level of erythropoietin declined rapidly, leucocytes normalized and, finally, even the excessively elevated platelet count returned to the normal range without any specific treatment.

Over the next 10 days her hemoglobin remained stable after transfusion of a total of four erythrocyte concentrates and her erythropoietin level rapidly declined. Serum iron was at the top of the normal range and the platelet count normalized rapidly without any treatment until the time of discharge from hospital. Therefore, also from the clinical course, ET was ruled out. After discharge from hospital the patient was prescribed oral iron supplementation (Ferro sanol duodenal^®^) to refill her iron storage pools. In the longer follow up her hemoglobin further increased to a level close to the normal range within the next month. The time course of hematological parameters measured during the stay in the hospital is summarized in Figure 2.

Despite the absence of typical clinical symptoms, histological examination of her duodenal mucosa showed typical signs of celiac disease of the infiltrative type, MARSH-Type I. At discharge from hospital, we recommended a gluten-free diet and the patient remained free of symptoms with a stable hemoglobin.

## Discussion

The clinical presentation of celiac disease is variable ranging from a "typical" form with diarrhea, steatorrhea, weight loss and impaired development, to forms with much more subtle symptoms or even a complete lack of typical clinical symptoms [3,6]. The patient in this case did not suffer from any typical signs of celiac disease but rather presented with signs of severe iron-deficiency anemia. No bleeding source could be detected despite appropriate diagnostic investigations and thus, in light of the extreme thrombocytosis, an underlying hematologic disease was suspected initially. However, bone marrow smear and bone marrow biopsy failed to provide support for the diagnosis of ET or another type of myeloproliferative disease. Furthermore, after transfusion of four erythrocyte concentrates, the platelet count completely normalized within two weeks. According to the WHO criteria, this rules out the possibility of ET since a sustained elevation of platelets of at least 600 × 10^3^/μl is mandatory for the diagnosis of ET [2].

Finally, the patient was diagnosed with celiac disease by small bowel biopsy. As summarized by Halfdanarson et al [3], anemia and/or thrombocytosis may occur as clinical manifestations of celiac disease in some cases [2,3]. Thrombocytosis in celiac disease could be secondary to inflammation, iron deficiency or functional hyposplenia [3]. In our case the patient had a severe iron deficiency anemia with a ferritin level below the assay range. Interestingly, as stated by Sanchez and Ewton [2], in the case of iron deficiency as the underlying cause for thrombocytosis, the platelet count rarely exceeds 700 × 10^3^/μl (see also [3]). Thus, the case presented here is of iron-deficiency anemia due to celiac disease with an unusually high platelet count, which to the best of the authors' knowledge, is the highest platelet count reported thus far in the literature.

Although there appears to be an association between iron-deficiency anemia and reactive thrombocytosis, the mechanism responsible is still a matter of debate [7-9]. Of interest in this respect is the notion that the amino acid sequence homology of thrombopoietin and erythropoietin might explain thrombocytosis in children with iron-deficiency anemia [8]. In our case, we observed a marked increase in the level of erythropoietin which declined rapidly after transfusion of four erythrocyte concentrates. Blood levels of erythropoietin are upregulated in response to anemia or arterial hypoxemia. Juxtatubular interstitial cells of the renal cortex sense oxygen levels through oxygen-dependent prolyl hydroxylase. This controls the expression of hypoxia-inducible factor 1α (HIF-1α), the transcription factor for erythropoietin [10]. In women with iron deficiency, Akan et al [9] reported elevated erythropoietin levels associated with thrombocytosis which both normalized after iron substitution. In the second subgroup of this study there was again a correlation between iron deficiency and erythropoietin levels; however, thrombocytosis in these patients was absent [9].

An elevation of platelet count was observed in animal studies and in patients with renal failure receiving erythropoietin as medication [11]. Erythropoietin and thrombopoietin belong to the same hematopoietic growth factor subfamily. Taken together, it is tempting to speculate that elevated erythropoietin levels in patients with iron-deficiency anemia lead to thrombocytosis as a result of some kind of cross-reactivity at the level of the thrombopoietin receptor c-mpl because of the homology of some amino acid sequences of erythropoietin to thrombopoietin [8]. The clinical course and correlation of erythropoietin and platelet counts in our patient would be in agreement with this hypothesis. However, recent *in vitro *data from Broudy et al [12] provide evidence to the contrary as they found no cross-competition for binding of erythropoietin and thrombopoietin to c-mpl and the erythropoietin receptor. On the other hand, it has been suggested that erythropoietin and thrombopoietin can synergistically stimulate megakaryocyte proliferation owing to signaling of erythropoietin at the level of bipotent erythroid/megakaryocyte progenitor cells [13,14]. Considering the data of Akan et al [9], the role of erythropoietin in thrombocytosis in patients with iron deficiency appears to be even more complex since not all patients with iron-deficiency anemia and elevated erythropoietin levels uniformly present with thrombocytosis. Thus, there has to be some kind of additional factor present in some iron-deficiency patients contributing to the stimulatory potential of erythropoietin on thrombopoesis.

In conclusion, even in the presence of an 'atypical' high platelet count one should consider the possibility of reactive thrombocytosis. The exact mechanism of thrombocytosis in iron-deficiency anemia remains to be defined. Cross-reaction between erythropoietin and thrombopoietin receptors owing to structural homology is discussed by some groups but this is contradicted by recent molecular data showing no cross-competition for binding of erythropoietin and thrombopoietin to c-mpl and the erythropoietin receptor. Recent data suggest a synergistic effect of erythropoietin and thrombopoietin on the level of bipotent erythroid/megakaryocyte progenitor cells. However, this fails to explain why not all patients with iron-deficiency anemia and elevated levels of erythropoietin present with thrombocytosis. Therefore, there must be additional, yet undefined mechanisms which contribute to the development of thrombocytosis in some patients with iron deficiency anemia.

## Competing interests

The author(s) declare that they have no competing interests.

## Authors' contributions

Drs. Voigt, Jordan, Sippel and Amoury equally contributed to the diagnosis and treatment of the patient, and contributed to drafting and revising the manuscript. Prof. Schmoll and Dr. Wolf critically revised the manuscript and gave final approval for publication.

## Consent

Written informed consent was obtained from the patient's next-of-kin for publication of this case report and accompanying images. A copy of the written consent is available for review by the Editor-in-Chief of this journal.
